# Cholesterol Stimulates the Transient Receptor Potential Melastatin 4 Channel in mpkCCD_c14_ Cells

**DOI:** 10.3389/fphar.2021.627875

**Published:** 2021-05-14

**Authors:** Yong-Xu Cai, Bao-Long Zhang, Miao Yu, Yan-Chao Yang, Xue Ao, Di Zhu, Qiu-Shi Wang, Jie Lou, Chen Liang, Liang-Liang Tang, Ming-Ming Wu, Zhi-Ren Zhang, He-Ping Ma

**Affiliations:** ^1^ Departments of Cardiology and Clinical Pharmacy, Harbin Medical University Cancer Hospital, Institute of Metabolic Disease, Heilongjiang Academy of Medical Science, Heilongjiang key laboratory for Metabolic disorder and cancer related cardiovascular diseases, and Key Laboratories of Education Ministry for Myocardial Ischemia Mechanism and Treatment, Harbin, China; ^2^ Department of Physiology, Emory University School of Medicine, Atlanta, GA, United States; ^3^ NHC Key Laboratory of Cell Transplantation, Harbin Medical University, Harbin, China

**Keywords:** TRPM4, Cholesterol, PI(4,5)P2, Lipid rafts, Lovastatin

## Abstract

We have shown that cholesterol regulates the activity of ion channels in mouse cortical collecting duct (CCD) mpkCCD_c14_ cells and that the transient receptor potential melastatin 4 (TRPM4) channel is expressed in these cells. However, whether TRPM4 channel is regulated by cholesterol remains unclear. Here, we performed inside-out patch-clamp experiments and found that inhibition of cholesterol biosynthesis by lovastatin significantly decreased, whereas enrichment of cholesterol with exogenous cholesterol significantly increased, TRPM4 channel open probability (*Po*) by regulating its sensitivity to Ca^2+^ in mpkCCD_c14_ cells. In addition, inside-out patch-clamp data show that acute depletion of cholesterol in the membrane inner leaflet by methyl-β-cyclodextrin (MβCD) significantly reduced TRPM4 *Po*, which was reversed by exogenous cholesterol. Moreover, immunofluorescence microscopy, Western blot, cell-surface biotinylation, and patch clamp analysis show that neither inhibition of intracellular cholesterol biosynthesis with lovastatin nor application of exogenous cholesterol had effect on TRPM4 channel protein abundance in the plasma membrane of mpkCCD_c14_ cells. Sucrose density gradient centrifugation studies demonstrate that TRPM4 was mainly located in cholesterol-rich lipid rafts. Lipid-protein overlay experiments show that TRPM4 directly interacted with several anionic phospholipids, including PI(4,5)P_2_. Depletion of PI(4,5)P_2_ with either wortmannin or PGE2 abrogated the stimulatory effects of exogenous cholesterol on TRPM4 activity, whereas exogenous PI(4,5)P_2_ (diC8-PI(4,5)P_2_, a water-soluble analog) increased the effects. These results suggest that cholesterol stimulates TRPM4 via a PI(4,5)P_2_-dependent mechanism.

## Introduction

The transient receptor potential melastatin 4 (TRPM4) channel is activated by an increase in intracellular Ca^2+^, which is permeable equally to Na^+^ and K^+^ (Wu et al., 2016; Ding et al., 2017). The channel has a relatively broad tissue expression pattern and its dysregulation is implicated in numerous diseases (Abriel et al., 2012). Recent studies have shown that TRPM4 mutations are associated with isolated cardiac conduction disease, right bundle-branch block, tachycardia, and Brugada syndrome (Kruse et al., 2009; Liu et al., 2010; Liu H. et al., 2013). Our previous study has shown that high salt diet-induced TRPM4 expression contributes to early stage endothelial damage in Dahl salt-sensitive hypertensive rat (Ding et al., 2017). We have also shown that TRPM4 channel is responsible for a Ca^2+^-activated nonselective cation current (NSC_Ca_) in CCD principal cells (Wu et al., 2016). Therefore, investigation of the regulation of TRPM4 would provide important information for many cellular functions which mediated by intracellular calcium.

Cholesterol, a major sterol in the mammalian plasma membrane, modulates the function of various ion channels (Levitan et al., 2010). Statins are potent inhibitors of 3-hydroxy-3-methylglutaryl-CoA reductase, the rate-limiting enzyme in the synthesis of cholesterol. We have shown that inhibition of cholesterol synthesis with lovastatin reduces the activity of epithelial sodium channel (ENaC) and that enrichment of cholesterol enhances activity of ENaC (Wei et al., 2007; Wang et al., 2009; Zhai et al., 2018). In contrast, inhibition of cholesterol synthesis with lovastatin stimulates the renal outer medullary K^+^ channel (ROMK) in mpkCCD_c14_ cells (Liu et al., 2015) and inwardly rectifying K^+^ channels in CHO cells (Romanenko et al., 2009). In addition, we have shown that lovastatin even antagonizes cyclosporine A (CsA)-induced cell apoptosis by reducing cholesterol synthesis in renal epithelial cells (Liu B. C. et al., 2013). However, it remains unclear how cholesterol regulates TRPM4 channels.

The cell membrane contains specialized microdomains referred to as lipid rafts which are enriched in cholesterol and sphingolipids. In lipid rafts, PI(4,5)P_2_ is thought to be localized in the inner leaflet of the plasma membrane, where it mediates many cellular functions (Balla, 2013). Previous studies have shown that PI(4,5)P_2_ is an important regulator of ion channels, including inward rectifier potassium channels (Huang et al., 1998), ENaC (Zhang et al., 2010), ROMK (Liu et al., 2014), and TRP channels (Braun, 2008). Depletion of cholesterol can cause PI(4,5)P_2_ diffusion from lipid rafts to non-lipid raft regions (Pike and Miller, 1998). Our previous studies show that inhibition of cholesterol synthesis by lovastatin elevates PI(4,5)P_2_ levels in non-lipid raft regions and stimulates ROMK channels which is located in non-lipid raft regions (Liu et al., 2015). Our recent studies also show that elevation of intracellular plasma cholesterol due to blockade of ABCA1 stimulates ENaC and contributes to CsA-induced hypertension (Wang et al., 2009; Wu et al., 2019). The follow-up study further shows that intracellular cholesterol stimulates ENaC in distal nephron cells by interacting with PI(4,5)P_2_ (Zhai et al., 2019). Previous studies also reveal that PI(4,5)P_2_ enhances TRPM4 activity via increasing the sensitivity to both membrane potential and [Ca^2+^]_i_ and depletion of PI(4,5)P_2_ causes desensitization of TRPM4 (Zhang et al., 2005). It has been suggested that R755 and R767 amino acids of TRPM4 N-terminus are the binding sites for potential regulatory molecules such as PI(4,5)P_2_ (Bousova et al., 2015). These studies together suggest that plasma membrane cholesterol may stimulate TRPM4 by promoting its interaction with PI(4,5)P_2_.

In the present study, we show that TRPM4 channel is localized in lipid rafts in mpkCCD_c14_ cells. We also show that enrichment of membrane cholesterol increases, whereas depletion of cholesterol by lovastatin decreases, TRPM4 activity by regulating its sensitivity to Ca^2+^ in mpkCCD_c14_ cells. Our results suggest that plasma membrane cholesterol stimulates TRPM4 via a PI(4,5)P_2_ dependent mechanism.

## Methods

### Cell Culture

The mpkCCDc_14_ line is an immortalized mouse collecting duct principal cell line, which was cultured as described previously (Bens et al., 1999). These cells were cultured in a 1:1 mixture of DMEM and Ham’s F-12 medium (GIBCO) supplemented with 20 mM HEPES, 2 mM L-glutamine, 50 nM dexamethasone, 1 nm triiodothyronine, 2% heat-inactivated FBS, and 0.1% penicillin-streptomycin. The mpkCCD_c14_ cells were plated at a density of 75,000 cells·cm^−1^ and grown on permeable supports to maintain cell polarization (Costar Transwells; 0.4 µm pore, 24 mm diameter) and cultured for at least 7 days prior to the experiments.

### Cell-Surface Biotinylation and Western Blot Assay

Biotinylation of the plasma membrane from mpkCCDc_14_ cells was performed as described previously (Wu et al., 2016). Briefly, after each treatment, the cells were incubated with a freshly prepared solution of 1.0 mg/ml EZ-Link sulfo-N-hydroxysuccinimide disulfide-biotin (Pierce, 21331) in borate buffer for 30 min at 4°C. The biotin reaction was quenched for 5 min with 0.1 mM lysine. An equal amount of lysate protein (1 mg) from each sample was respectively incubated with 50 µl of immobilized streptavidin-agarose beads (Pierce, 20349) at 4°C for overnight with gentle shaking. The beads were washed four times with RIPA buffer. Equal amounts of samples from either whole-cell or biotinylated plasma membranes were loaded and separated by a 10% SDS-polyacrylamide gel and transferred to polyvinylidene fluoride membranes. The membranes were then blocked in 5% non-fat dry milk for 1 h, followed by incubation with rabbit polyclonal anti-TRPM4 antibody (1:200 dilution; Alomone Labs; ACC-044) at 4°C for overnight. Rabbit polyclonal anti-GAPDH (1:1000 dilution; Santa cruz; sc-25778) was used as internal controls. Bands were visualized with enhanced chemiluminescence (Bio-Rad, Cat. No., 170-5061) and quantified via densitometry using the ImageJ software (NIH ImageJ software).

### Sucrose Gradient Assay

Lipid raft fractionation was isolated as described previously (Liu et al., 2015). Briefly, mpkCCDc14 cells suspension were homogenized in 0.5% Brij 96V (Sigma)/TNEV buffer (10 mM Tris-HCl, pH 7.5; 150 mM NaCl; 5 mM EDTA; 2 mM Na vanadate; and protease inhibitor cocktail) on ice for 30 min. The supernatant (500 μl) was mixed with an equal volume of 80% sucrose in TNEV and transferred into a centrifuge tube (13 × 51 mm; Beckman Coulter, Palatine, IL, United States). Three milliliters of 35% sucrose in TNEV was carefully layered on top of the mixture, followed by another 1 ml layer of 5% sucrose. The sucrose gradient was then centrifuged in a SW 50.1 rotor (Beckman Coulter) at 34,000 rpm (∼ 110,000 g) for 20 h at 4°C. After centrifugation, fractions were collected starting from the top to bottom of the tube. Thirteen fractions (∼ 400 μl) were collected, and equal volumes of each fraction were analyzed by 10% gradient SDS-PAGE and immunoblotted with TRPM4 antibodies and caveolin-1 (1:1000 dilution; Cell Signaling Tech cat# 3267S).

### Patch-Clamp Recording

Single-channel currents were recorded from mpkCCD_c14_ cells under the voltage-clamp mode with an Axopatch-200B amplifier (Molecular Devices, Sunnyvale, CA), using inside-out patch-clamp configurations, as described previously (Wu et al., 2016). Data were acquired and sampled with a low-pass, 1 kHz, eight-pole Bessel filter using a Digidata 1440A analog-digital interface (Axon Instruments, Inc.). The mpkCCDc_14_ cells were thoroughly washed with NaCl solution containing (in mM) 145 NaCl, 1 MgCl_2_, 1 CaCl_2_, 10 glucose, and 10 HEPES, adjusted pH to 7.4 with NaOH. This NaCl solution was used for filling the bath in the patch chamber and filling the patch pipette. The patch pipette was pulled with Borosilicate glass, giving a tip resistance of 5–8 MΩ when filled with NaCl solution. Single-channel currents were obtained at a holding potential of 80 mV for inside-out recordings, and only the patches with the seal resistance >2 GΩ were used. Experiments were conducted at room temperature (22–25°C). Prior to analysis, the single-channel traces were further filtered at 100 Hz. The single-channel amplitude was constructed by all-point amplitude histogram and the histograms were fit using multiple Gaussians and optimized using a simplex algorithm. *P*_*O*_ was calculated as *P*_*O*_ = *NP*_*O*_/*N*, where N (N was estimated by the current amplitude histogram during at least 5 min recording period when the channel was maximally activated by Ca^2+^) is the number of active channels in the patch. According to a total of 20 inside-out patches, we found that the channel activity can be maximally activated after we excised the patch membrane and exposed the inner leaflet of the membrane to 1 mM Ca^2+^ in the bath. Under the condition, we recorded more than 5 min and the channel activity remained very high. This maximal activation of the channel after we excised the patch membrane into a bath solution containing 1 mM Ca^2+^ was also used for the estimation of N. We also switched to a solution containing 10 mM EGTA without any calcium to mark the zero current levels. More importantly, since lovastatin significantly inhibited the TRPM4 open probability by reducing its sensitivity to Ca^2+^. In order to obtain the number of active channels in the patches, single channel current was recorded from inside-out parches exposed the patch membrane to the bath containing 5 mM CaCl_2_, followed by a bath solution with 10 mM EGTA without any calcium. The free Ca^2+^ concentration after chelating CaCl_2_ with EGTA was determined using free Web software Winamac (Stanford University, Stanford, CA, United States), as previously described (Wu et al., 2014).

### Confocal Microscopy

Confocal microscopy experiments were performed as previously reported (Wu et al., 2016). Briefly, after fixation with 4% paraformaldehyde at room temperature for 10 min, the cells were permeabilized with 0.2% Triton X-100 in PBS for 10 min and blocked with 5% BSA/PBS-T for 30 min. Rabbit polyclonal anti-TRPM4 antibody (alomone ACC-044; 1:100 dilution) was added in 1% BSA/TBS-T for overnight at 4°C. The sections were washed in TBS-T and incubated with Alexa Fluor 488 conjugated donkey anti-rabbit IgG (Invitrogen A21206, 1:1000 dilution) and Alexa-594-conjugated cholera toxin B (CTB) (Invitrogen C34777) for 1 h. All slides were imaged using a confocal microscope (Olympus, Fluoview1000, Japan). To detect cholesterol levels in the plasma membrane of mpkCCDc_14_ cells, the cells were incubated with 5 μg/ml filipin (Sigma, Cat#: F9765) for 30 min. Filipin staining was viewed by confocal microscope using DAPI filter. The control fluorescent intensity is used as a calibrator, and relative fluorescent intensity is calculated against this calibrator. All slides were imaged using a confocal microscope (Olympus, Fluoview1000, Japan) and analyzed using Olympus Fluoview FV1000 version 3.1 software. Identical acquisition settings were used for all images. To quantify colocalizations, the image analysis program ImageJ was used. Both Pearson and Manders coefficients were calculated.

### Lipid-Protein Overlay

To test the lipid-binding properties of TRPM4, Protein lipid overlay assays were performed using PIP Strips from Invitrogen (Chicago, IL, United States) as previously described (Zhang et al., 2010). The strip is a piece of nitrocellulose membrane on which 15 phospholipids at 100 pM and a blank sample were loaded by the manufacturer. Briefly, strips were blocked in TBS-Tween (0.1%, TBS-T) and 3% BSA for 1 h. The mpkCCDc_14_ cells lysate was then diluted in the blocking buffer to 0.5 μg·ml^−1^ and incubated overnight at 4°C. To detect the possible binding of TRPM4 to spotted phospholipids, the Strips were incubated with rabbit polyclonal antibodies directed against TRPM4 (1:200; ACC-044; Alomone Labs, Jerusalem, Israel) similar to the western blot method.

### Chemicals

All chemicals for electrophysiological recordings were purchased from Sigma-Aldrich (St Louis, MO, United States) except when specified. DiC8-PI(4,5)P_2_ was purchased from Echelon Biosciences. Both wortmannin and prostaglandin E2 (PGE2) were used to pre-treat mpkCCDc_14_ cells for 30 min for patch-clamp experiments.

### Data Analysis.

Data are reported as mean values ±SEM. Statistical analysis was performed with GraphPad Prism 5 software (GraphPad; La Jolla, CA) was used for all statistical calculations. Student *t* test was used between two groups. Analysis of variance was used for multiple comparisons. Results were considered significant if *P* < 0.05**.**

## Results

### Inhibition of Cholesterol Biosynthesis Decreases, Whereas Enrichment of Cholesterol Increases, Transient Receptor Potential Melastatin4 Channel Activity by Regulating its Sensitivity to Ca^2+^ in mpkCCD_c14_ Cells.

**FIGURE 1:**
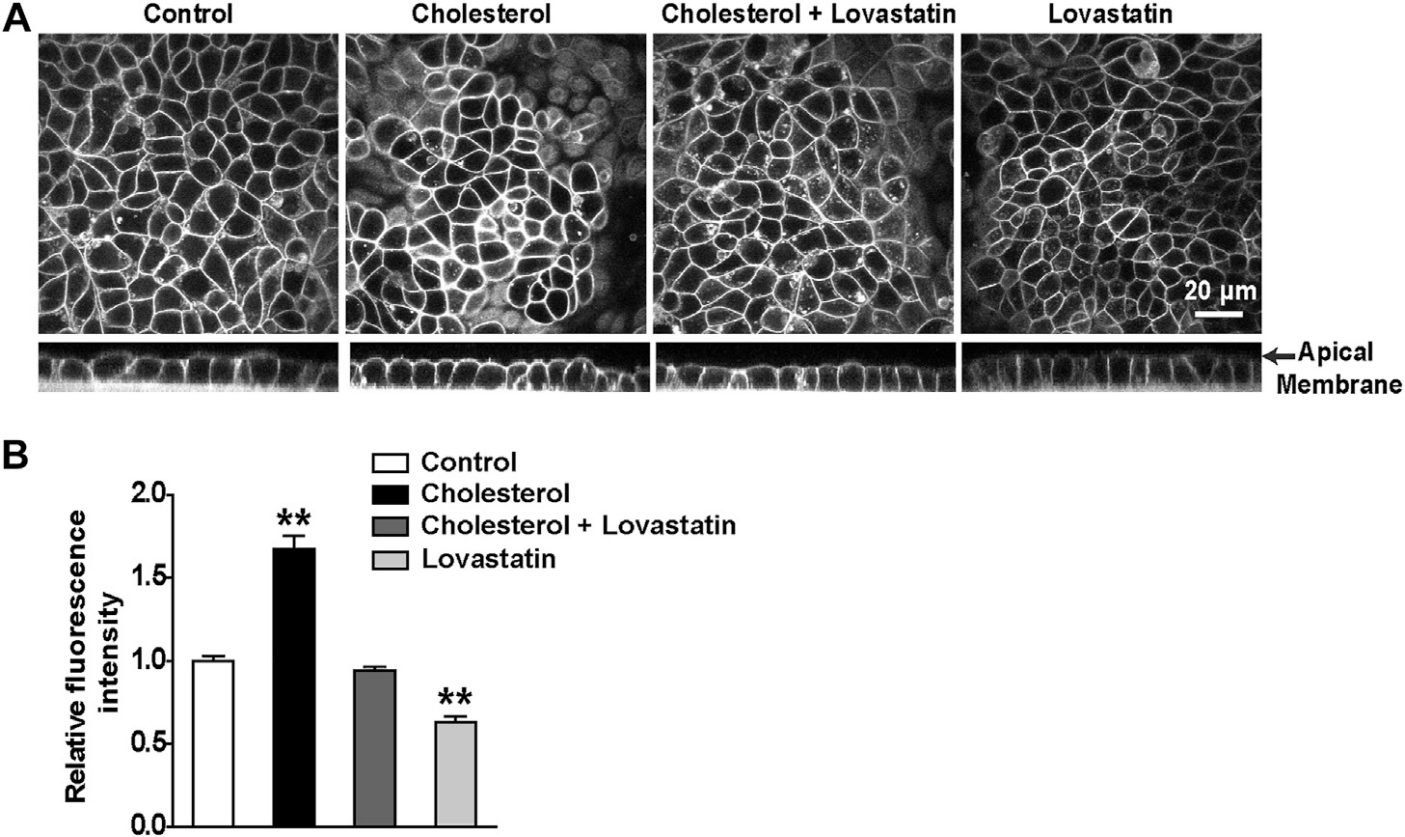
Treatment of cells with cholesterol increases, but with lovastatin decreases, membrane cholesterol levels in mpkCCD_c14_ Cells. **(A)** Representative confocal microscopy images of mpkCCD_c14_ cells stained with filipin either from XY optical sections near the apical membrane (*top*) or from XZ optical sections (*bottom*). **(B)** Summary plots of fluorescence intensity of cholesterol levels under each indicated conditions. Cells were either under control conditions or treated for 48 hrs with 30 μg/ml cholesterol alone, 30 μg/ml cholesterol plus 5 μM lovastatin, or 5 μM lovastatin alone, respectively. Data are from 18 images in three sets of separate experiments. **P < 0.01, significantly different with control.

**FIGURE 2:**
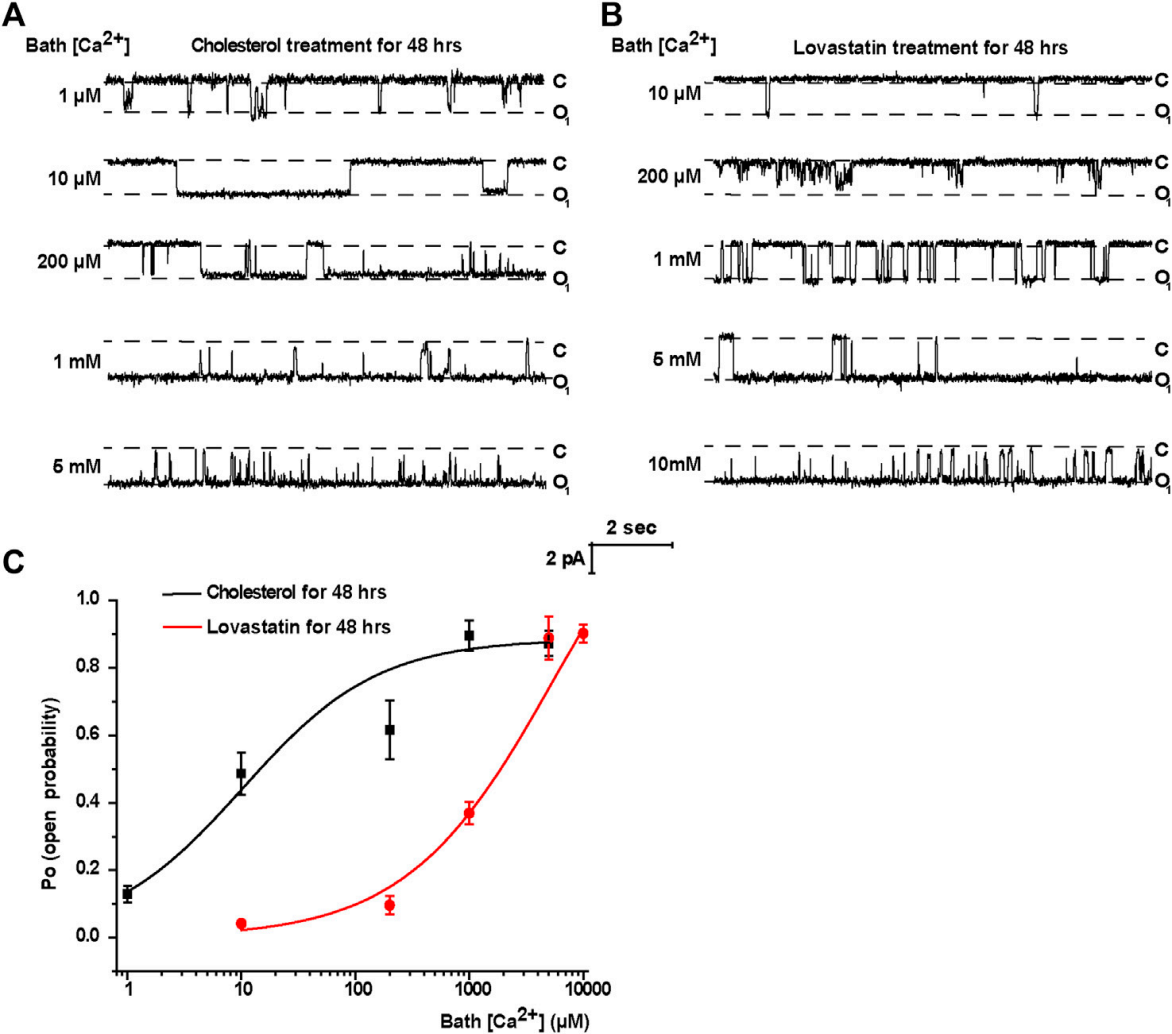
Treatment of cells with cholesterol increases, but with lovastatin decreases, TRPM4 channel activity by regulating its sensitivity to Ca^2+^ in mpkCCD_c14_ Cells. **(A)** Representative single channel recording from inside-out patches exposed the patch membrane to the bath containing different concentrations of free Ca^2+^. The cells were treated with 30 μg/ml cholesterol for 48 hrs. From top to bottom: 1 μM, 10 μM, 200 μM, 1 mM, and 5 mM free bath Ca^2+^. “C” indicates channel at the closed state; “O” indicates single-level openings. **(B)** Representative single channel recording from inside-out patches exposed the patch membrane to the bath containing different concentrations of free Ca^2+^. The cells were treated with 5 μM lovastatin for 48 hrs. From top to bottom: 10 μM, 200 μM, 1 mM, 5 mM and 10 mM free bath Ca^2+^. “C” indicates channel at the closed state; “O” indicates single-level openings. **(C)** The effect of membrane cholesterol on Ca^2+^-dependence of channel opening. Channel *P*_O_ was plotted as a function of free Ca^2+^ concentration in the bath. *P*o values are shown for patches either with exogenous cholesterol treatment (black line) or lovastatin treatment (red line). *n* = 4–7 cells for different data points.

To manipulate the plasma membrane cholesterol content, lovastatin and exogenous cholesterol were used as we previously described (Song et al., 2014). The mpkCCD_c14_ cells were treated with 5 μM lovastatin, 30 μg/ml exogenous cholesterol, or 5 μM lovastatin plus 30 μg/ml exogenous cholesterol for 48 hrs. Then, plasma membrane cholesterol levels in mpkCCD_c14_ cells were evaluated by filipin staining. The data show that exogenous cholesterol significantly increased, whereas lovastatin significantly decreased, the cholesterol levels in the plasma membrane of mpkCCDc_14_ cells, and that co-treatment of the cells with both lovastatin and exogenous cholesterol did not alter cholesterol levels (Figures 1A,B). To further determine whether these treatments affect TRPM4 channel activity by regulating its sensitivity to Ca^2+^, the channel activity was recorded by exposing the patch membrane to the bath containing different concentrations of free Ca^2+^ using the inside-out patch-clamp technique. We have previously demonstrated that the concentration of Ca^2+^ required for 50% of maximal activation of TRPM4 (EC50) was ∼32.6 μM under basal conditions without manipulation of membrane cholesterol levels (Wu et al., 2016). Here, we found that the EC50 after reducing membrane cholesterol with lovastatin was only ∼5.76 mM and that this effect was significantly reversed to ∼10.2 μM by enrichment of membrane cholesterol with exogenous cholesterol (Figure 2). These results indicate that elevation of membrane cholesterol increases the TRPM4 channel activity by enhancing its sensitivity to Ca^2+^ in mpkCCD_c14_ cells.

### Exogenous Cholesterol Restores the Inhibition of Transient Receptor Potential Melastatin4 by Deletion of Membrane Cholesterol With Methyl-β-Cyclodextrin in mpkCCD_c14_ Cells

**FIGURE 3:**
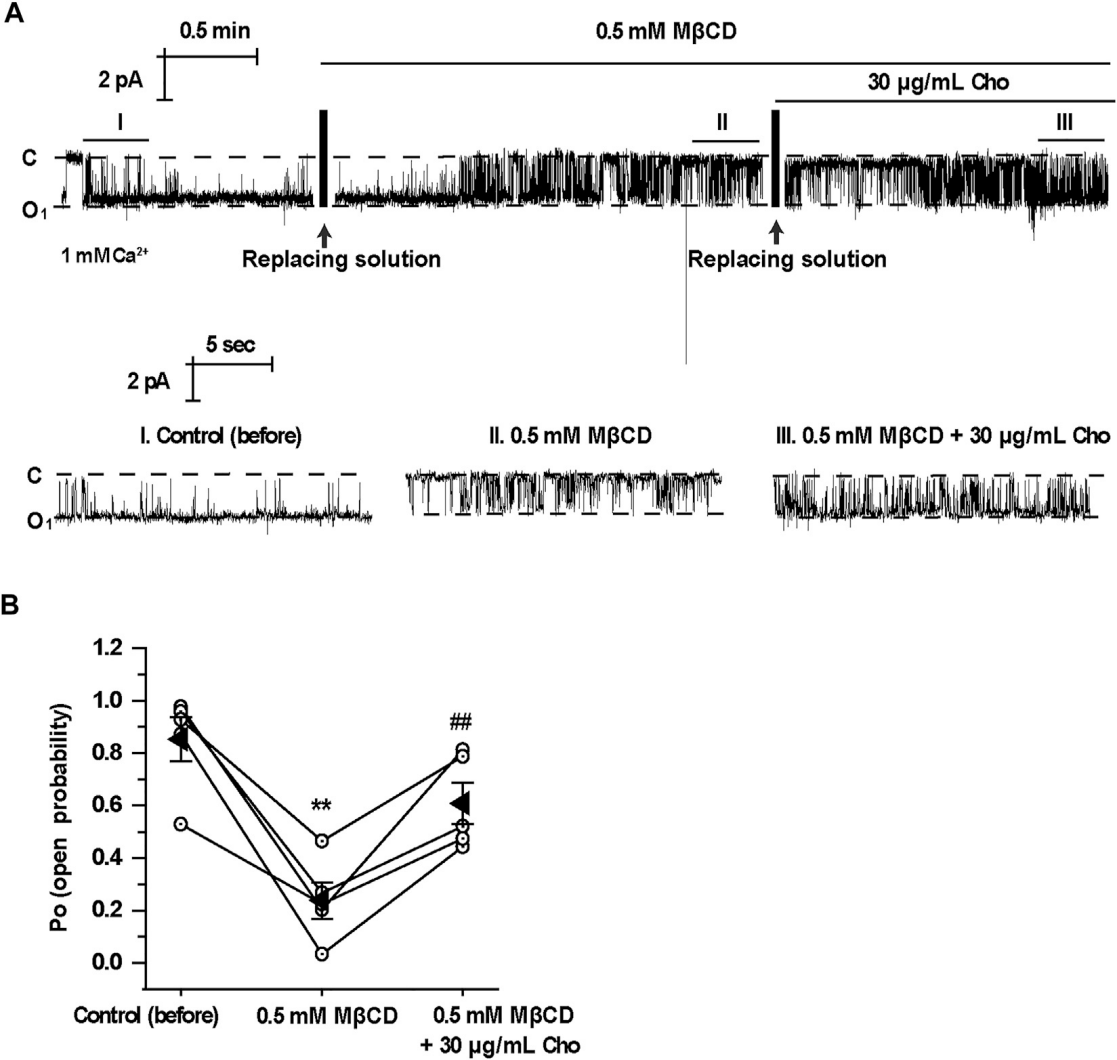
Application of MβCD to the cytoplasmic bath decreases TRPM4 channel activity which is reversed by exogenous cholesterol. **(A)** A representative single channel recording from an inside-out patch shows TRPM4 activity before and after replacement of control cytoplasmic bath solution first with a solution containing 0.5 mM MβCD and then with a solution containing 30 μg/ml cholesterol. “C” indicates channel at the closed state. “O” indicates single-level openings. “I, II, and III” are zoom-ins of the single-channel recording. **(B)** Summary plots of TRPM4 channel *P*_*O*_ under each indicted condition. *n* = 5 paired experiments, **P < 0.01, significantly different with control; ^##^ *P* < 0.01, significantly different with cells treated with 0.5 mM MβCD.

To further determine whether acute depletion of cholesterol with MβCD affects TRPM4 channel activity, we performed excised inside-out patch-clamp experiments in mpkCCD_c14_ cells. Here, we found that acute extraction of cholesterol out of the inner leaflet of the patch membrane with MβCD significantly reduced TRPM4 Po and that application of exogenous cholesterol reversed the reduction of TRPM4 channel activity induced by MβCD (Figures 3A,B). These data together suggest that cholesterol in the inner leaflet of the plasma membrane is required for maintaining TRPM4 activity.

### Enrichment of Plasma Membrane Cholesterol Stimulates Transient Receptor Potential Melastatin4 via a PI(4,5)P_2_-Dependent Mechanism

**FIGURE 4:**
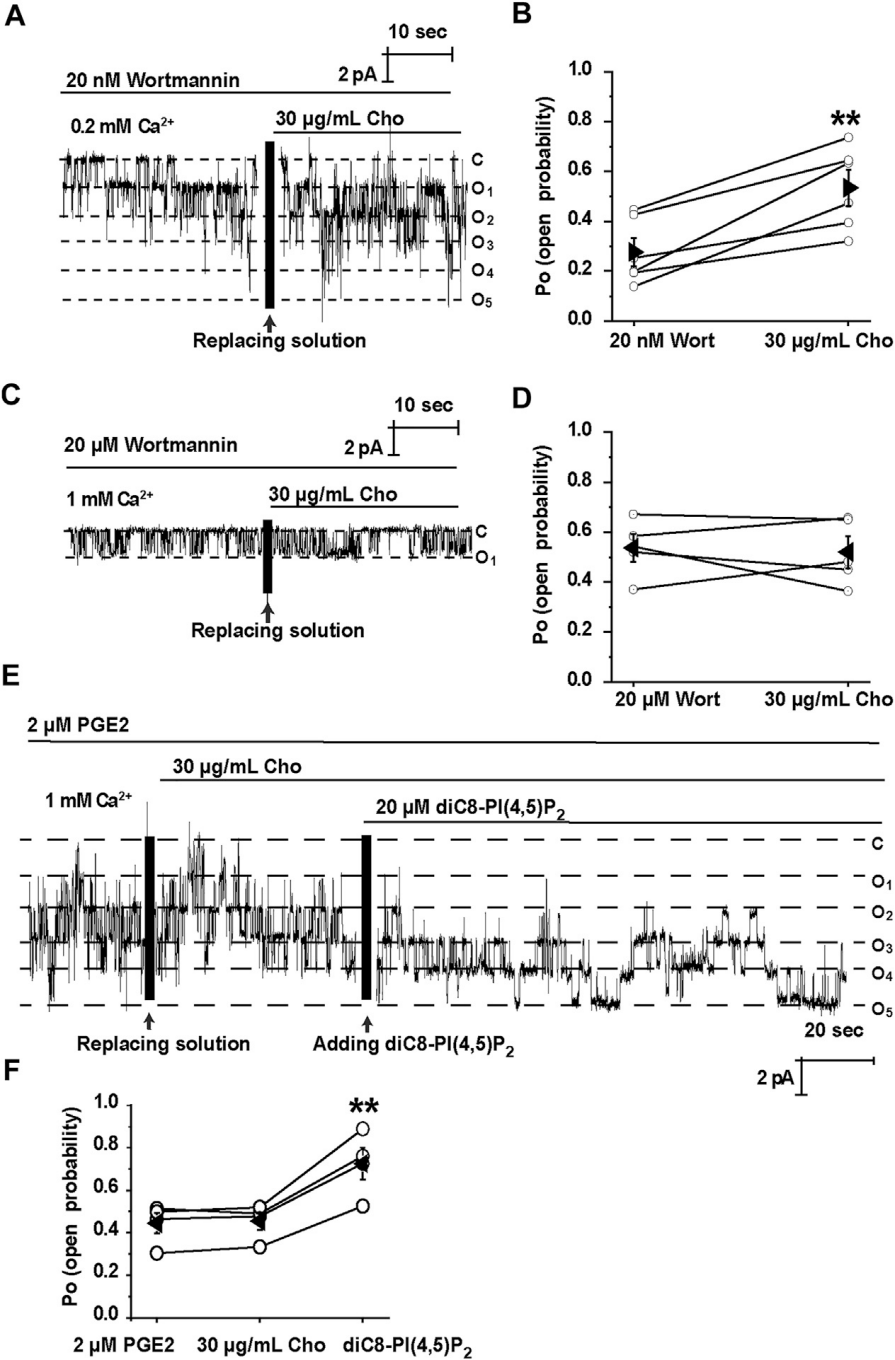
Cholesterol stimulates TRPM4 via a PI(4, 5)P_2_-dependent mechanism. **(A)** A representative single channel recording shows that treatment of mpkCCD_c14_ cells with 20 nM wortmannin had no effect on cholesterol-induced TRPM4 channel activity. **(B)** Summary plots of TRPM4 channel *P*_*O*_ under each indicated conditions. *n* = 6 paired experiments, **P < 0.01, significantly different with cells treated with 20 nm wortmannin. **(C)** A representative single channel recording shows that depletion of PI(4, 5)P_2_ with 20 μM wortmannin abrogated cholesterol-induced TRPM4 channel activity. **(D)** Summary plots of TRPM4 channel *P*_*O*_ under indicated conditions. *n* = 5 paired experiments. **(E)** A representative single channel recording shows that application of 20 μM diC8-PI(4,5)P_2_ induced TRPM4 channel activity after cholesterol failed to stimulate TRPM4 in the presence of 2 μM PGE2. **(F)** Summary plots of TRPM4 channel *P*_*O*_ under indicated conditions. *n* = 4 paired experiments. **P < 0.01, significantly different with cells treated with 2 μM PGE2 or 30 μg/ml cholesterol.

We have previously shown that cholesterol regulates ROMK channels by altering PI(4,5)P_2_ localization (Liu et al., 2015). It is known that PI(4,5)P_2_ stimulates TRPM4 channels (Nilius et al., 2006). Therefore, we would ask whether PI(4,5)P_2_ is required for cholesterol to stimulate TRPM4. Wortmannin, at high concentrations, is a PI4K inhibitor, therefore preventing the synthesis of PI(4,5)P_2_ and depleting membrane PI(4,5)P_2_ (Saleh et al., 2009). Our data show that depletion of PI(4,5)P_2_ by wortmannin (20 μM) abrogated exogenous cholesterol-induced TRPM4 channel activity (Figures 4A,B). In contrast, application of 20 nM wortmannin, which is unable to alter PI(4,5)P_2_ levels, had no effects on cholesterol-induced TRPM4 channel activity (Figures 4C,D). Since PGE2 depletes PI(4,5)P_2_ via activation of Gq-coupled EP1 receptors (Harraz et al., 2018), PGE2 (2 μM) was also used to examine whether cholesterol can stimulate TRPM4 without PI(4,5)P_2_. Our data showed that PGE2 abolished the stimulatory effects of exogenous cholesterol on TRPM4 activity, whereas exogenous PI(4,5)P_2_ (diC8-PI(4,5)P_2_, a water-soluble analog) increased the effects (Figures 4E,F). These data suggest that elevation of plasma membrane cholesterol stimulates TRPM4 via a PI(4,5)P_2_-dependent mechanism.

### PI(4,5)P_2_ Binds to Transient Receptor Potential Melastatin4 Channels in mpkCCD_c14_ Cells

**FIGURE 5:**
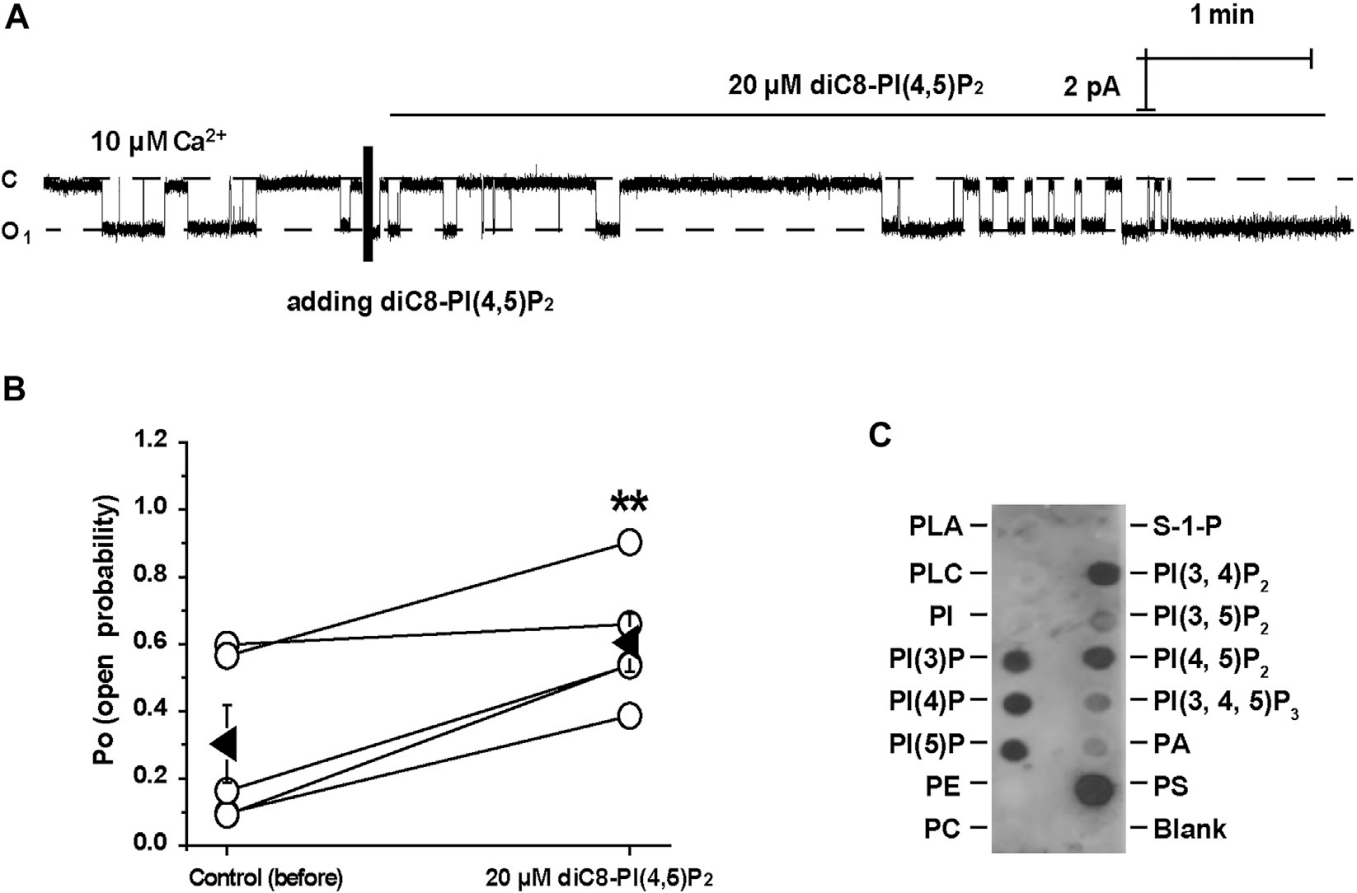
PI(4, 5)P_2_ stimulates TRPM4 via a physical interaction. **(A)** A representative single channel recording shows that diC8-PI(4,5)P_2_ significantly increased TRPM4 activity. **(B)** Summary plots of TRPM4 channel *P*_*O*_ under each indicated conditions. *n* = 5 paired experiments. **P < 0.01, significantly different with control. **(C)** TRPM4 was detected in dots where most anionic phospholipids including PI(4, 5)P_2_ are located on PIP Strips membrane. Data represent three individual experiments showing consistent results.

Previous studies have demonstrated that PI(4,5)P_2_ is a strong positive modulator of TRPM4 (Nilius et al., 2006). Consistently, our inside-out data show that diC8-PI(4,5)P_2_ (20 μM) significantly increased the TRPM4 activity (Figures 5A,B). To further determine whether TRPM4 can bind to PI(4,5)P_2_, lipid-protein overlay experiments were performed by using PIP Strips. Our data show that TRPM4 physically binds to almost all phosphatidylinositols (PI) including PI(4,5)P_2_ (Figure 5). These data suggest that PI(4,5)P2 stimulates TRPM4 channels probably via a physical interaction.

### Transient Receptor Potential Melastatin4 is Mainly Located in Cholesterol-Rich Lipid Rafts in mpkCCD_c14_

**FIGURE 6:**
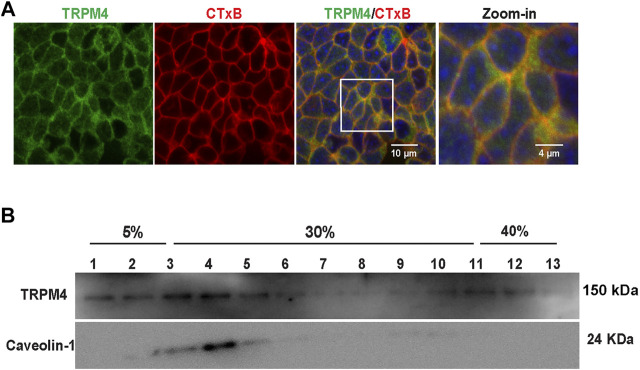
TRPM4 channels are mainly located in lipid rafts. **(A)** Representative confocal microscopy images indicate that majority of TRPM4 (green) was co-localized with cholera toxin B (red) in the apical membrane; white rectangular box indicate zoomed-in areas shown in the Zoom-in panels. Data represent five individual experiments showing consistent results **(B)** Majority of TRPM4 was detected in low-density regions in sucrose gradient experiments; Caveolin-1 was used as a control protein that is known to be located in lipid rafts. Data represent three individual experiments showing consistent results.

Our previous studies have shown that cholesterol in lipid rafts maintains PI(4,5)P_2_ in lipid rafts (Liu et al., 2015). To determine whether TRPM4 is also located in lipid rafts to physically interact with PI(4,5)P_2_, we labeled lipid rafts with fluorescence-tagged cholera toxin (CTX) and TRPM4 with its specific antibody. Quantitative analysis with ImageJ showed that TRPM4 channel was colocalized with lipid rafts (Pearson coefficient was 0.726 ± 0.049 and Manders coefficients were 0.902 ± 0.042 [M_1_], red color and 0.932 ± 0.037 [M_2_], green color). Confocal microscopy data showed that TRPM4 channel was co-localized with lipid rafts (Figure 6A). Consistently, the data from sucrose density gradient assays also show that fractions 2–5 are denoted as the lipid raft fractions as indicated by caveolin-1, a marker of membrane lipid rafts and TRPM4 was mainly enriched in lipid raft membranes in mpkCCD_c14_ cells (Figure 6B). These data indicate that plasma membrane cholesterol stimulates TRPM4 by holding PI(4,5)P_2_ in lipid rafts.

### Changes in Plasma Membrane Cholesterol Have No Effect on Transient Receptor Potential Melastatin4 Expression.

**FIGURE 7:**
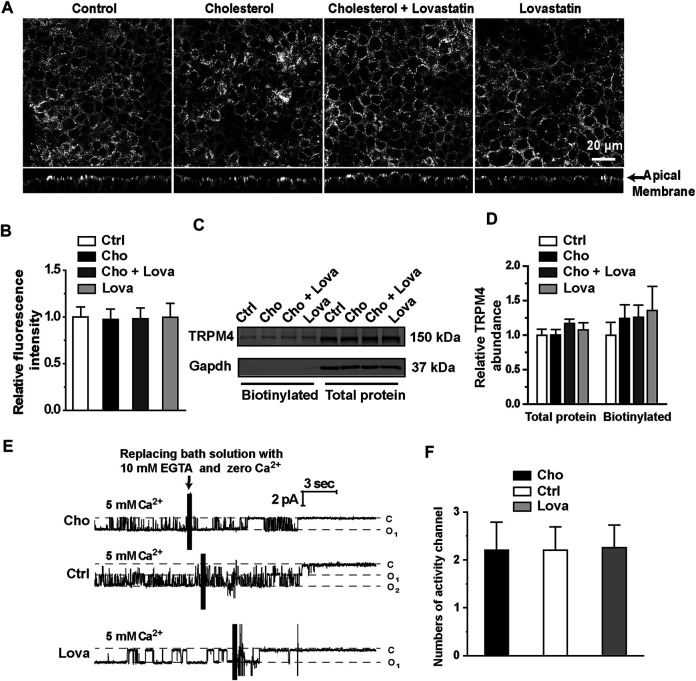
Treatment with cholesterol or lovastatin does not alter expression levels of TRPM4 in mpkCCDc14 Cells. **(A)** Representative confocal microscopy images of mpkCCDc14 cells stained with TRPM4 antibody under each indicated conditions. **(B)** Summary plots of fluorescence intensity of TRPM4. Data are from 24 cells in four sets of separate experiments. **(C)** Representative Western blots from cell-surface biotinylated and the total proteins of TRPM4 protein. **(D)** Summary plots of relative expression of TRPM4. Cells were either under control conditions or treated with 30 μg/ml cholesterol alone, 30 μg/ml cholesterol plus 5 μM lovastatin, or 5 μM lovastatin alone for 48 hrs, respectively. *n* = 5. **(E)** Representative single channel recording from inside-out patches exposed the patch membrane to the bath containing 5 mM CaCl_2_, followed by a bath solution with 10 mM EGTA and no calcium. Cells were either under control conditions or treated with 30 μg/ml cholesterol, or 5 μM lovastatin for 48 hrs, respectively. **(F)** Summary plots of the number of active channels in the patches under each indicated condition. *n* = 5 for control cells, *n* = 5 for cells treated with cholesterol, *n* = 4 for cells treated with lovastatin.

Since plasma membrane cholesterol could regulate TRPM4 activity, we examined whether manipulation of cholesterol levels will affect the expression of TRPM4 in mpkCCD_c14_ cells. Confocal microscopy experiments were performed using control cells and cells treated for 48 hrs with 30 μg/ml exogenous cholesterol, 5 μM lovastatin, or 5 μM lovastatin plus 30 μg/ml exogenous cholesterol. These data showed that enrichment of cholesterol or inhibition of cholesterol biosynthesis has no effect on TRPM4 expression in mpkCCDc14 cells (Figures 7A,B). To confirm the results from confocal microscopy experiments, Western blot and cell-surface biotinylation assay data also showed that the total and membrane levels of TRPM4 in mpkCCD_c14_ cells were unaltered by cholesterol enrichment or inhibition of cholesterol biosynthesis (Figures 7C,D). To further examine whether manipulating the membrane cholesterol of mpkCCD_c14_ cells can affect TRPM4 cell membrane abundance, the number of active channels was recorded by exposing the patch membrane to the bath containing 5 mM CaCl_2,_ followed by a bath solution with 10 mM EGTA and no calcium. Our data showed that neither cholesterol enrichment nor inhibition of cholesterol biosynthesis affects the number of active channels in the patches from mpkCCD_c14_ cells (Figures 7E,F). Thus, the potentiating effect of cholesterol on TRPM4 activity cannot be attributed to an enhanced its surface expression.

## Discussion

The present study shows that pharmacological approaches to manipulate the plasma membrane cholesterol content regulate TRPM4 channel activity in mpkCCD_c14_ cells. Inhibition of cholesterol biosynthesis decreases, whereas enrichment of cholesterol in cell membrane increases, TRPM4 channel activity by regulating its sensitivity to Ca^2+^ in mpkCCD_c14_ cells. Our results suggest that elevation of plasma membrane cholesterol stimulates TRPM4 via a PI(4,5)P_2_ dependent mechanism.

Different mechanisms have been proposed to account for cholesterol regulation of ion channels. Several lines of evidence suggest that cholesterol may directly regulate ion channels by binding to specific sites of the channels or indirectly regulate ion channels by promoting the interaction with intracellular signal cascades including PI(4,5)P_2_. Here we show that TRPM4 is located in lipid rafts where cholesterol is located. However, we also show that cholesterol no longer stimulates TRPM4 channels when PI(4,5)P_2_ is depleted by inhibition of phosphatidylinositol 5-kinase with a high concentration of wortmannin, indicating that cholesterol does not directly stimulate TRPM4 channels. Our previous studies have demonstrated that PI(4,5)P_2_ is co-localized with cholesterol in the microvilli where a majority of lipid rafts is located (Zhai et al., 2018; Zhai et al., 2019) and that inhibition of cholesterol synthesis reduces PI(4,5)P_2_ in the microvilli by causing PI(4,5)P_2_ diffusion into planar regions (Liu et al., 2015). Therefore, decreases in membrane cholesterol would decrease the activity of TRPM4 channels which is located in lipid rafts by reducing PI(4,5)P_2_ which is also located in lipid rafts. Conversely, increases in membrane cholesterol would increase the activity of TRPM4 channels by elevating PI(4,5)P_2_. We also favor the nation that TRPM4 is located in the lipid rafts and that exogenous cholesterol acts as a shuttle to collect free PI(4,5)P_2_ in non-lipids to translocate PI(4,5)P_2_ into lipid rafts to stimulate TRPM4.

Since it is known that PI(4,5)P_2_ sensitizes TRPM4 to Ca^2+^ (Zhang et al., 2005), in the experiments we used wortmannin and PGE2 to reduce PI(4,5)P_2_, however, in order to achieve a basal activity we increased the concentration of Ca^2+^ (1 mM) before we applied cholesterol. As shown in Figures 4C,D, under the condition that PI(4,5)P_2_ was reduced and Ca^2+^ was elevated, cholesterol failed to increase TRPM4 activity. The failure should not be due to a saturated activation of the channel by 1 mM Ca^2+^, because additional PI(4,5)P_2_ still elevated the channel activity (Figure 4E). Although our data suggest that the effect of cholesterol on TRPM4 channel activity is PI(4,5)P_2_-dependent. we cannot rule out the possibility that TRPM4 can directly interact with cholesterol, because lipids may well be coordinated in the channel complex and interact by allosteric linkage. Indeed, previous studies have suggested that TRPM4 channel contains putative cholesterol binding sites (Autzen et al., 2018). However, our data suggest that it is unlikely that enrichment of cholesterol stimulates TRPM4 activity by direct interaction with the channel via the cholesterol-binding sites, because cholesterol no longer stimulates TRPM4 channels when PI(4,5)P_2_ is depleted. We argue that the direct interaction between cholesterol and TRPM4 may only play a role in maintaining TRPM4 localization in lipid rafts.

Our previous report suggests that TRPM4 accounts for the nonselective cation channel activity found in the CCD principal cells (Wu et al., 2016). However, it still remains unclear whether TRPM4 channel activity is responsible for K^+^ secretion and Na^+^ reabsorption under physiological conditions, because it requires a high concentration of intracellular Ca^2+^ to activate TRPM4 channels. Based on our findings that endogenous TRPP2 and TRPV4 assemble to form a non-selective calcium-permeable channel complex in the CCD principal cells (Zhang et al., 2013). Activation of TRPP2/TRPV4 would allow Ca^2+^ influx to generate sufficient magnitude (μM) in the subapical membrane to activate TRPM4 channels. Therefore, the present study indicates that TRPM4 channel activity is responsible for K^+^ secretion and Na^+^ reabsorption under physiological conditions. Since cholesterol accumulation causes kidney dysfunction and contributes to hypertension, the stimulation of TRPM4 by elevated cholesterol would have pathophysiological significance.

## Conclusion

Our data show that TRPM4 channel is localized in lipid rafts in mpkCCD_c14_ cells. In addition, enrichment of membrane cholesterol increases, whereas deletion of cholesterol by lovastatin decreases, TRPM4 activity by regulating its sensitivity to Ca^2+^ in mpkCCD_c14_ cells. Our data also suggest that plasma membrane cholesterol stimulates TRPM4 via a PI(4,5)P_2_ dependent mechanism.

## Data Availability

The data that support the findings of this study are available from the corresponding author upon reasonable request.

## Abbreviations

TRPM4, transient receptor potential melastatin 4; CCD, cortical collecting duct; MβCD, bethyl-β-cyclodextrin; ENaC, epithelial sodium channel; NSCCa, Ca2+-activated nonselective cation current; ROMK, renal outer medullary K+ channel; CsA, cyclosporine A.
